# Generation of novel genetically modified rats to reveal the molecular mechanisms of vitamin D actions

**DOI:** 10.1038/s41598-020-62048-1

**Published:** 2020-03-30

**Authors:** Miyu Nishikawa, Kaori Yasuda, Masashi Takamatsu, Keisuke Abe, Kairi Okamoto, Kyohei Horibe, Hiroki Mano, Kimie Nakagawa, Naoko Tsugawa, Yoshihisa Hirota, Tetsuhiro Horie, Eiichi Hinoi, Toshio Okano, Shinichi Ikushiro, Toshiyuki Sakaki

**Affiliations:** 1 Department of Biotechnology, Faculty of Engineering, Toyama Prefectural University, 5180 Kurokawa, Imizu, Toyama 939-0398 Japan; 2 Department of Pharmaceutical Engineering, Faculty of Engineering, Toyama Prefectural University, 5180 Kurokawa, Imizu, Toyama 939-0398 Japan; 3 Department of Hygienic Sciences, Kobe Pharmaceutical University, 4-19-1 Motoyamakita-machi, Higashinada-ku, Kobe 658-8558 Japan; 4 Department of Health and Nutrition, Faculty of Health and Nutrition, Osaka Shoin Women’s University, 4-2-26 Hishiya-nishi, Higashi-Osaka, 577-8550 Japan; 5 Laboratory of Biochemistry, Faculty of Bioscience and Engineering, College of Systems Engineering and Science, Shibaura Institute of Technology, 307 Fukasaku, Minuma-ku, Saitama 337-8570 Japan; 6 Laboratory of Pharmacology, Department of Bioactive Molecules, Gifu Pharmaceutical University, Gifu, Japan; 7 United Graduate School of Drug Discovery and Medical Information Sciences, Gifu University, Gifu, Japan

**Keywords:** Calcium and vitamin D, Osteogenesis imperfecta

## Abstract

Recent studies have suggested that vitamin D activities involve vitamin D receptor (VDR)-dependent and VDR-independent effects of 1α,25-dihydroxyvitamin D_3_ (1,25(OH)_2_D_3_) and 25-hydroxyvitamin D_3_ (25(OH)D_3_) and ligand-independent effects of the VDR. Here, we describe a novel *in vivo* system using genetically modified rats deficient in the *Cyp27b1* or *Vdr* genes. Type II rickets model rats with a mutant *Vdr* (R270L), which recognizes 1,25(OH)_2_D_3_ with an affinity equivalent to that for 25(OH)D_3_, were also generated. Although *Cyp27b1*-knockout (KO), *Vdr-*KO, and *Vdr* (R270L) rats each showed rickets symptoms, including abnormal bone formation, they were significantly different from each other. Administration of 25(OH)D_3_ reversed rickets symptoms in *Cyp27b1*-KO and *Vdr* (R270L) rats. Interestingly, 1,25(OH)_2_D_3_ was synthesized in *Cyp27b1*-KO rats, probably by Cyp27a1. In contrast, the effects of 25(OH)D_3_ on *Vdr* (R270L) rats strongly suggested a direct action of 25(OH)D_3_ via VDR-genomic pathways. These results convincingly suggest the usefulness of our *in vivo* system.

## Introduction

The active form of vitamin D_3_, 1α,25-dihydroxyvitamin D_3_ (1,25(OH)_2_D_3_), plays important roles in osteogenesis, calcium homeostasis, cellular differentiation, and immune responses^1^. 1,25(OH)_2_D_3_ is generated by two hydroxylation steps from vitamin D_3_: C-25 hydroxylation by hepatic CYP2R1 and CYP27A1 and subsequent 1α-hydroxylation by renal 1α-hydroxylase (CYP27B1)^2^. The vitamin D receptor (VDR) mediates the genomic action of active vitamin D_3_. Binding of active vitamin D_3_ to the VDR triggers its heterodimerization to the retinoid X receptor and subsequent translocation to the nucleus. This translocation results in regulating target gene expression by formation of the VDR complex on vitamin D-responsive elements in the promoter regions of target genes, such as osteocalcin and osteopontin in bones and the calcium channels and calbindins in intestines^3^.

CYP24A1, one of the well-known vitamin D target genes, is involved in inactivating 1,25(OH)_2_D_3_ through sequential metabolism that starts with C-24 or C-23 hydroxylation of 1,25(OH)_2_D_3_^4^. A variety of vitamin D derivatives have been developed as drugs for rickets, osteoporosis, psoriasis, secondary hyperparathyroidism, and chronic kidney disease. Because all of these compounds show high affinity for the VDR, these pharmacological actions are considered to be VDR mediated. However, as with 1,25(OH)_2_D_3_, they might also have non-VDR-mediated actions. Thus, pharmacological action studies of vitamin D derivatives are essential for future drug discovery.

Recent reports have demonstrated that 25(OH)D_3_ can regulate gene expression by binding directly to the VDR^5–9^. We have reported previously that 25(OH)D_3_ is a potential VDR ligand in immortalized human prostate PZ-HPV-7 cells^10^. Whereas the affinity of 25(OH)D_3_ for VDR is more than 100-fold lower than that of 1,25(OH)_2_D_3_^11^, the plasma concentration of 25(OH)D_3_ (vitamin D binding protein (DBP)-bound form) is several hundred-fold higher than that of 1,25(OH)_2_D_3_ (DBP-bound form). Based on its *K*_d_ value for the VDR and the plasma concentration of 25(OH)D_3_, these biological and biochemical findings suggest that 25(OH)D_3_ could be a physiologically important agonist of the VDR.

To confirm the direct action of 25(OH)D_3_ *in vivo*, we previously examined its effect on osteogenesis in *Cyp27b1* knockout (KO) mice. These mice have no detectable 1,25(OH)_2_D_3_ in their plasma and exhibit all the hallmarks of type I rickets, such as reduced bone mineral density and hypocalcemia. Surprisingly, 1,25(OH)_2_D_3_ was detected at normal levels in *Cyp27b1*-KO mice administered 25(OH)D_3_ at 150 μg•kg^−1^•day^−1^. Plasma calcium levels, bone mineral densities, and sexual reproduction in *Cyp27b1*-KO mice were all normalized by 25(OH)D_3_ administration, while plasma 25(OH)D_3_ levels were enhanced several-fold relative to the normal level^12^. Based on the activity of 1α-hydroxylation toward 25(OH)D_3_ in liver mitochondrial fractions prepared from *Cyp27b1*-KO mice, we assumed that Cyp27a1 converted 25(OH)D_3_ to 1,25(OH)_2_D_3_^12^.

The body size and blood volume of mouse models appear to be too small to perform pharmacokinetic studies of vitamin D and its analogs. Furthermore, it is difficult to analyze the small organs in such models. Thus, we generated a *Cyp27b1*-deficient rat model to clarify the detailed metabolism of 25(OH)D_3_. In the current study, *Cyp27b1*-KO rats were produced by genome editing using the CRISPR/Cas9 system^13–15^. As mentioned above, it was difficult to verify the direct effect of 25(OH)D_3_ in *Cyp27b1-*KO mice due to the Cyp27b1-independent production of 1,25(OH)_2_D_3_, and it was reasonable to assume that a similar phenomenon would occur in rats. Therefore, we also generated genetically modified (GM) rats with a mutant Vdr (R270L), which corresponds to human VDR (R274L) derived from patients with type II rickets^16^. Notably, mutant Vdr (R270L) has approximately 1000-fold reduced VDR affinity toward 1,25(OH)_2_D_3_ due to the substitution of Arg for Leu at position 270, which is responsible for binding the 1α-hydroxyl group of 1,25(OH)_2_D_3_^17^. Because mutant Vdr (R270L) recognizes 1,25(OH)_2_D_3_ with lower affinity than that for 25(OH)D_3_, *Vdr* (R270L) rats have none of the high-affinity ligands in their bodies. Hence, the conversion from 25(OH)D_3_ to 1,25(OH)_2_D_3_ has almost no effect on Vdr-mediated actions in *Vdr* (R270L) rats. We also simultaneously obtained *Vdr*-KO rats as a side product of *Vdr* (R270L) rat production.

In the current study, we elucidated the relationships among vitamin D_3_ metabolism, calcium homeostasis, and osteogenesis using these GM rats. We also suggest that 25(OH)D_3_ administration may be useful in treating both type I and type II rickets.

## Results

### Generation of GM rats

Five offspring were obtained from 125 embryos microinjected for *Cyp27b1*-KO, 4 of which had mutations at target sites. Among the 4 pups with target mutations, 1 was found to have a 25 amino acid deletion containing the cysteine residue, which is the fifth ligand of heme iron and the active center of Cyp27b1. This founder was used in this study (#1 in Supplementary Fig. S1b).

The *Vdr* sequences of 74 of 109 offspring obtained from 311 embryos microinjected for *Vdr* (R270L) were analyzed. Two of these pups were found to have the mutant *Vdr* in one chromosome, resulting from homology-directed repair with coinjected ssODNs. The mutant *Vdr* showed that the WT arginine codon at position 270 (CGC) was substituted by a leucine (CTC) codon (#1 and #45 in Supplementary Fig. S2b). Heterozygosity was demonstrated by the presence of peaks for both G and T at the mutation site (see Supplementary Fig. S2c). By contrast, 6 pups had indel mutations resulting from nonhomologous end joining, 1 of which had a stop codon, TGA, at position 266 resulting from a frameshift mutation (V266STOP) (#26 in Supplementary Fig. S2b). Human mutant VDR (Y295STOP) has been reported as a VDR-deficient mutation^16^; thus, the corresponding mutant *Vdr* (V266STOP) was used as a founder for the *Vdr*-knockout model.

We also confirmed that no off-target site (OTS) events occurred in potential OTSs searched by the CRISPR Direct tool (http://crispr.dbcls.jp/) among all GM rat strains except for *Cyp27b1*-OTS8. We could not examine the OTS event in *Cyp27b1*-OTS8 because it was impossible to amplify this region (see Supplementary Tables S1 and S2).

### Appearance and growth of GM rats

**Figure 1:**
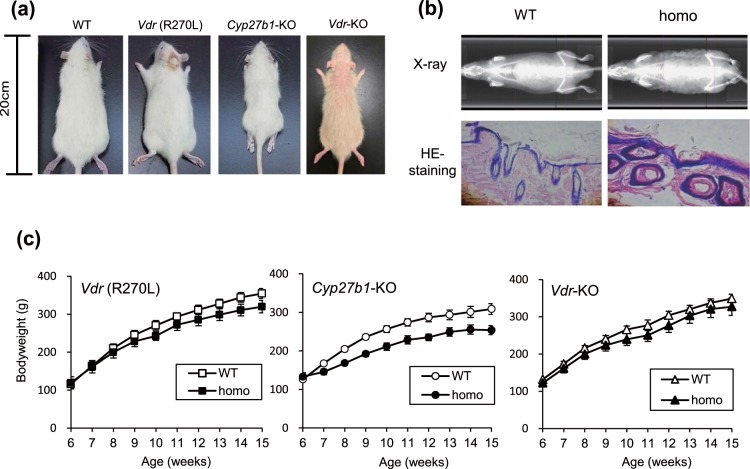
The appearance and growth of GM rats. (**a**) Comparison of body size and skin phenotype at 15 weeks of age. (**b**) Details of the skin phenotype in *Vdr*-KO rats. Upper panels, X-ray images of the whole body; lower panels, H&E staining of the dorsal skin. (**c**) Growth curves from 6 to 15 weeks of age. WT male Wistar rats in (**a**) were commercially obtained from Sankyo Labo Service Corporation Inc. (Tokyo, Japan). WT rats in (**b,c**) were generated in-house by the mating of the heterozygotes of each strain. The values are shown as the means ± SEMs (n = 3–5 animals/group).

The phenotypes of GM rats are summarized in Table 1. The appearance of WT, mutant *Vdr* (R270L) and *Vdr-*KO rats fed an F-2 diet and *Cyp27b1*-KO rats fed a CE-2 diet at 15 weeks after birth are detailed in Fig. 1a. Whereas *Cyp27b1-*KO rats were much smaller than WT rats, *Vdr* (R270L) and *Vdr-*KO rats were somewhat smaller than WT rats. *Vdr-*KO rats had abnormal skin with alopecia (Fig. 1a), which was also reported in *Vdr-*KO mice^18^ and human type II rickets^16^. In addition to hair loss, elasticity and softness of the skin was markedly decreased, resulting in the skin of *Vdr*-KO rats appearing wavy (Fig. 1b, upper panels). H&E staining of the dorsal skin of *Vdr-*KO rats demonstrated decreased follicles and increased keratinization and cyst formation (Fig. 1b, lower panels). Heterozygotes of *Cyp27b1*-KO, *Vdr* (R270L), and *Vdr-*KO rats showed phenotypes similar to those of WT rats at 15 weeks.

**Table 1:** Summary of the phenotype among GM rats at 15 weeks of age.

Strain	Diet	Growth	Femur formation	BMD	Alopecia	Plasma Ca	PTH	1,25(OH)_2_D_3_
WT	F-2 or CE-2^a^	→	normal	→	no	→	→	→
*Vdr* (R270L)	F-2	↓	abnormal	→	no	↓	↑	↑↑
*Cyp27b1*-KO	CE-2	↓↓	abnormal	↓	no	↓↓	↑	↓
*Vdr*-KO	F-2	↓	abnormal	→	yes	→	↑	↑↑

^a^WT rats from heterozygotes of *Vdr* (R270L) or *Vdr*-KO rats were maintained with the F-2 diet. WT rats from *Cyp27b1*-KO heterozygote rats were maintained with a CE-2 diet. The dietary components of F-2 and CE-2 are shown in Supplementary Tables S11 and S12.

Growth curves of mutant *Vdr* (R270L) and *Vdr-*KO rats fed the F-2 diet and *Cyp27b1-*KO rats fed the CE-2 diet are shown in Fig. 1c. Growth was significantly inhibited in *Cyp27b1*-KO rats but was only slightly inhibited in *Vdr* (R270L) and *Vdr-*KO rats compared to that of WT rats (Fig. 1c). Notably, 4 of 7 male *Cyp27b1*-KO rats fed the F-2 diet containing 0.74% calcium  died prior to 9 weeks of age, and none survived to 10 weeks of age (data not shown), whereas no animals died at 15 weeks of age among the *Cyp27b1*-KO rats fed the CE-2 diet. Thus, subsequent analyses were performed with the F-2 diet for mutant *Vdr* (R270L) and *Vdr-*KO rats, while a CE-2 diet containing 1.15% calcium was used for *Cyp27b1*-KO rats.

### Osteogenesis and bone metabolism-related parameters in blood

**Figure 2:**
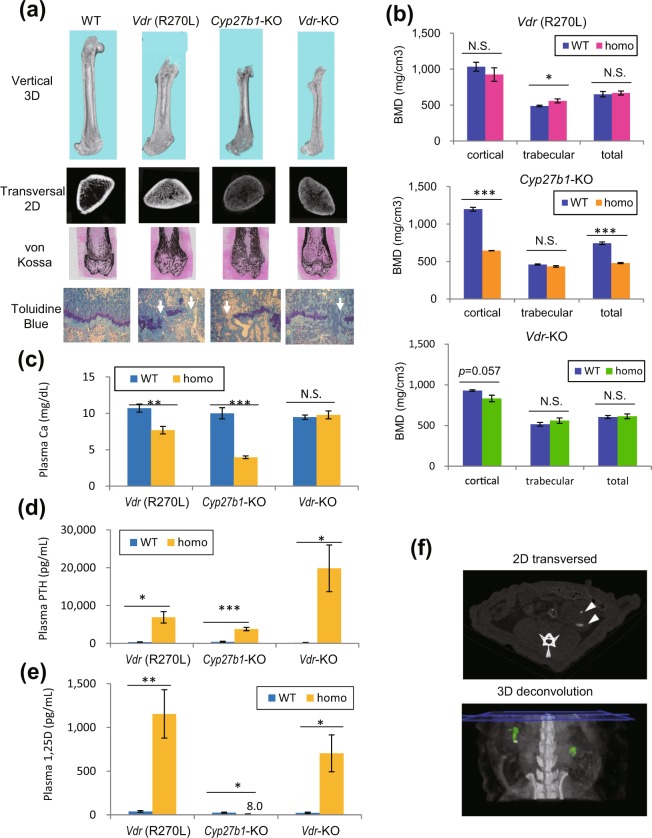
Bone malformation and abnormal bone metabolism parameters in plasma. (**a**) Phenotypes of femora. Top panels, 3D μ-CT images of femora; second panels, 2D μ-CT images of horizontal distal femur sections; third panels, von Kossa staining of distal femora; bottom panels, toluidine blue staining of epiphyseal cartilage. White arrows indicate fractures of the epiphyseal plate. (**b**) BMD of the cortical, trabecular, and total bones at the distal femur. The values are shown as the means ± SEMs (n = 4–5 animals/group). (**c–e**) Plasma concentrations of calcium (Ca) (**c**), PTH (**d**) and 1,25(OH)_2_D (1,25D)(**e**). The values are shown as the means ± SEMs (n = 4–8, n = 3–8 and n = 4–7 animals/group for Ca, PTH and 1,25D levels, respectively). (**f**) Abdominal μ-CT images in *Vdr*-KO rats at 25 weeks of age. Upper panel, 2D transverse image. Lower panel, 3D deconvolution image. Arrowheads and green colored regions indicate ectopic calcification in the kidney. *p < 0.05, **p < 0.01, ***p < 0.001, and N.S.: not significant by Student’s t-test.

The top and second panels of Fig. 2a show 3D-reconstituted images of the femur with a vertical section and a 2D-horizontal scan image at the middle region of the femur analyzed by μ-CT, respectively. The femur lengths of *Cyp27b1-*KO, mutant *Vdr* (R270L) and *Vdr-*KO rats were shorter than those of WT rats. CT scanning and von Kossa staining of femurs showed hyperplasia of trabecular bones, with narrowed medullary cavities, in all GM rats. Although *Vdr* (R270L) and *Vdr-*KO rats did not show clear changes in total bone mineral density (BMD), the BMD of cortical bone in *Cyp27b1*-KO rats was significantly decreased (t = 21.108, df = 3.245, *p* < 0.001), resulting in decreased total BMD (t = 13.782, df = 6, *p* < 0.001) (Fig. 2a, third panels and Fig. 2b).

Marked cartilaginous disorganization was seen in the growth plates of *Cyp27b1*-KO rats, *Vdr* (R270L) rats and *Vdr*-KO rats at 15 weeks of age, as evidenced by toluidine blue staining, indicating the histological features of rickets (Fig. 2a and Supplementary Fig. S8). In addition, increased unmineralized osteoid, which is the hallmark of osteomalacia, was observed in *Cyp27b1*-KO rats and *Vdr* (R270L) rats but not in *Vdr-*KO rats at 15 weeks of age. Although all GM rats showed abnormal bone morphology, *Cyp27b1*-KO rats showed more severe bone disorders than the other GM rats (Figs. 1 and 2). Rickets model mice, including *Cyp27b1-*KO and *Vdr-*KO mice, have significantly lower plasma calcium  levels than WT mice^19^. As expected, the plasma calcium level was significantly reduced in *Vdr* (R270L) rats (t = 3.881, df = 12, *p* = 0.002) and *Cyp27b1*-KO rats (t = 7.584, df = 7.845, *p* < 0.001) (Fig. 2c). The level of parathyroid hormone (PTH), whose secretion is induced by the reduced plasma calcium level via calcium-sensing receptor (CaSR) in the parathyroid, was significantly increased in *Vdr* (R270L) rats (t = 4.295, df = 5.026, *p* = 0.008) and *Cyp27b1*-KO rats (t = 8.448, df = 8, *p* < 0.001) (Fig. 2d). Surprisingly, the plasma calcium level in *Vdr*-KO rats was normal at 15 weeks (t = 0.579, df = 8, *p* = 0.578), whereas the plasma calcium level in *Vdr*-KO mice was much lower than that in wild-type mice^18,19^. However, time course analysis of plasma calcium and PTH levels in *Vdr*-KO rats revealed that the plasma calcium level was reduced and that the plasma PTH level was higher than that in WT rats at 8 and 10 weeks (see Supplementary Fig. S7a,b). The plasma calcium level in *Vdr*-KO rats was increased to normal levels by 15 weeks. However, the plasma PTH level in *Vdr*-KO rats was not reduced (a significant trend, with t = 2.252, df = 5.002, *p* = 0.074), suggesting hyperparathyroidism in these rats. In addition, plasma concentrations of phosphorus and creatinine were elevated in some *Vdr-*KO rats at 25 weeks, suggesting kidney dysfunction (values marked with # in Supplementary Fig. S7e,f). μCT analysis revealed kidney stone formation in *Vdr-*KO rats at 25 weeks of age, with high plasma P and creatinine (Fig. 2f). These results suggest that the *Vdr*-KO rats appear to exhibit tertiary hyperparathyroidism. This type of hyperparathyroidism occurs as a result of long-term secondary hyperparathyroidism.

**Figure 3:**
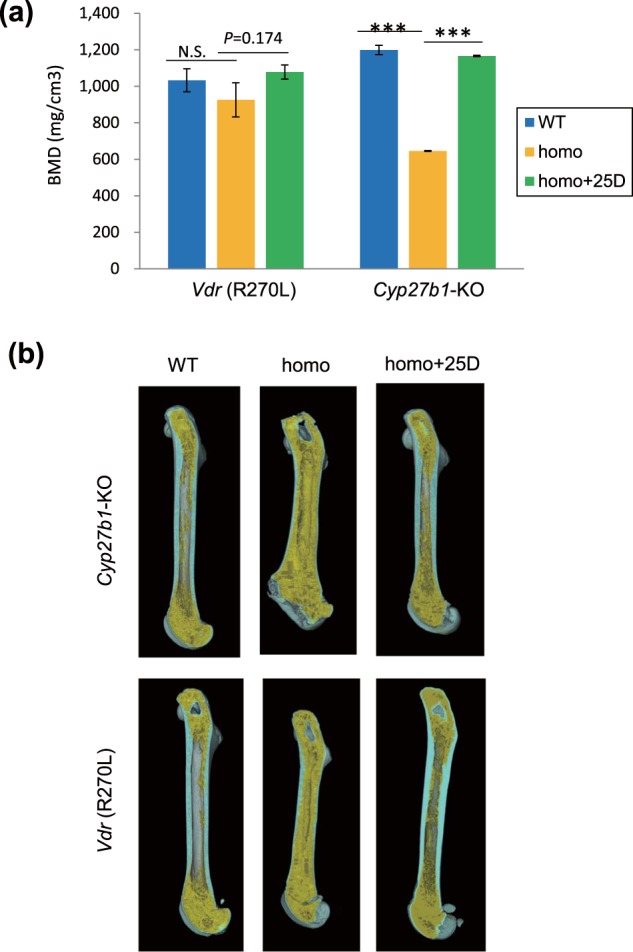
Effect of 25(OH)D_3_ on osteogenesis in *Vdr*(R270L) and *Cyp27b1*-KO rats. (**a**) BMD of the cortical bone in the distal femur. The values are shown as the means ± SEMs (n = 3–5 animals/group). (**b**) 3D deconvolution μ-CT images of femur vertical section. Cortical and trabecular bones are colored light blue and yellow, respectively. ***p < 0.001 and N.S.: not significant by two-way ANOVA.

### Plasma concentration of 1,25(OH)_2_D_3_

As shown in Fig. 2e, the plasma 1,25(OH)_2_D_3_ level was significantly increased in *Vdr* (R270L) (t = 4.024, df = 8, *p* = 0.004) and *Vdr-*KO rats (t = 2.723, df = 5, *p* = 0.042) but was significantly decreased in *Cyp27b1*-KO rats (8.0 ± 3.2 pg/mL (mean ± SEM, n = 7)) compared to that in WT rats (24.8 ± 5.2 pg/mL, (mean ± SEM, n = 7)) (t = 2.771, df = 12, *p* = 0.017). This result was somewhat different from that in *Cyp27b1*-KO mice^12^, which showed no detectable 1,25(OH)_2_D_3_ (<5 pg/mL).

### Effects of 25(OH)D_3_ administration on type I rickets in *Cyp27b1-* KO rats

In a previous study, we demonstrated that dietary administration of 25(OH)D_3_ to *Cyp27b1*-KO mice reversed type I rickets hallmarks, such as growth failure, skeletal disorders and hypocalcemia^12^. Dietary administration of 25(OH)D_3_ to *Cyp27b1*-KO rats at 200 μg•kg^−1^•day^−1^ also significantly reversed growth failure (see Supplementary Fig. S8a,b). The BMD of the cortex and trabecular bone were normalized by 25(OH)D_3_ administration (Fig. 3a), resulting in structural normalization of the femur (Fig. 3b, upper panels). Histological analysis of the femoral sections demonstrated a normalized structure of the cortex and trabecular bone by 25(OH)D_3_ administration in *Cyp27b1*-KO rats. Disruption of the growth plate and chondrocytes was also normalized by 25(OH)D_3_ administration (see Supplementary Fig. S8c). In addition, 25(OH)D_3_ administration dramatically corrected the osteomalacic features of the *Cyp27b1*-KO rats (see Supplementary Fig. S9).

**Figure 4:**
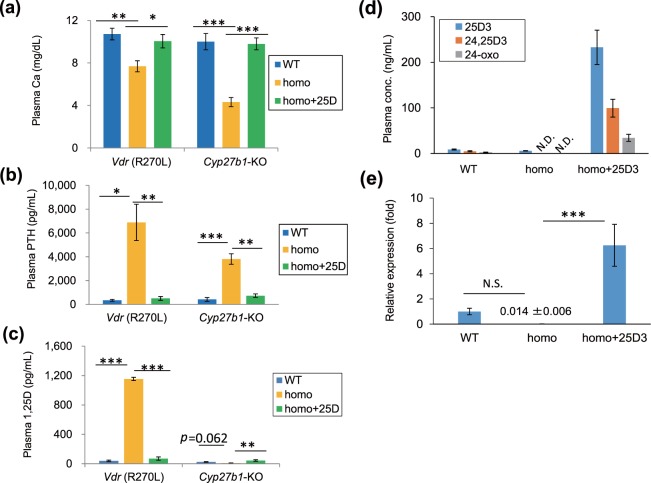
Effect of 25(OH)D_3_ on bone metabolism parameters in *Vdr* (R270L) and *Cyp27b1*-KO rats. (**a–c**) Plasma concentrations of calcium(Ca) (**a**), PTH (**b**) and 1,25(OH)_2_D (1,25D) (**c**) in *Vdr* (R270L) and *Cyp27b1*-KO rats. The values are shown as the means ± SEMs (n = 6–8, n = 4–8 and n = 5–7 animals/group for plasma Ca, PTH and 1,25D levels, respectively). (**d**) Plasma concentration of 25(OH)D_3_ and its Cyp24a1-dependent metabolites in *Vdr* (R270L) rats. The values are shown as the means ± SEMs (n = 4–5 animals/group). ND: less than 1.0 nM. (**e**) Relative expression of renal *Cyp24a1* mRNA in *Vdr* (R270L) rats. The values are shown as the means ± SEMs (n = 4–5 animals/group). N.S.: not significant, *p < 0.05, **p < 0.01, and ***p < 0.001 by two-way ANOVA.

The plasma calcium level of *Cyp27b1*-KO rats was fully restored by 25(OH)D_3_ administration (F(1, 21) = 45.6, *p* < 0.001), and the markedly elevated plasma PTH level in *Cyp27b1*-KO rats (F(1, 11) = 90.6, *p* < 0.001) was normalized after 25(OH)D_3_ administration (F(1, 11) = 62.2, *p* < 0.001) (Fig. 4a,b).

As expected, plasma 1,25(OH)_2_D_3_ deficiency in *Cyp27b1*-KO rats was normalized by 25(OH)D_3_ administration (F(1, 16) = 15.4, *p* = 0.001) (Fig. 4c). This result was in accordance with previous results from *Cyp27b1*-KO mice fed 25(OH)D_3_^12^. Liver mitochondrial fractions prepared from *Cyp27b1*-KO rats showed 1α-hydroxylation activity toward 25(OH)D_3_. This activity was inhibited by fadrozole, which is a potent inhibitor of Cyp27a1 (see Supplementary Fig. S10). These results were also quite similar to those in *Cyp27b1-*KO mice, suggesting that the most likely candidate for the other 1α-hydroxylase is Cyp27a1^12^.

### Effects of 25(OH)D_3_ administration on type II rickets in *Vdr* (R270L) rats

25(OH)D_3_ treatment of *Vdr* (R270L) rats clearly normalized these bone disorders, with increased cortical BMD (*p* = 0.174) in these rats (Fig. 3a and lower panels in Fig. 3b). The decreased plasma calcium in *Vdr* (R270L) rats (F(1, 19) = 12.5, *p* = 0.002) was normalized by the 25D-F-2 diet (F(1, 19) = 7.5, *p* = 0.013) (Fig. 4a). In correspondence to the reduction in the plasma calcium level, the elevated plasma PTH (F(1, 12) = 20.0, *p* = 0.001) and 1,25(OH)_2_D_3_ (F(1, 12) = 24.1, *p* < 0.001) contents were reduced to normal levels in *Vdr* (R270L) rats fed the 25D-F-2 diet (F(1, 12) = 16.8, *p* = 0.001 for PTH, and F(1, 12) = 22.8, *p* < 0.001 for 1,25(OH)_2_D_3_) (Fig. 4b,c).

Figure 4d shows plasma concentrations of 25(OH)D_3_ and its two metabolites formed by Cyp24a1, 24,25(OH)_2_D_3_ and 24-oxo-25(OH)D_3_. The plasma concentration of 25(OH)D_3_ in *Vdr* (R270L) rats fed the 25D-F-2 diet was approximately 500 nM, which is more than 20 times higher than that in WT rats fed the F-2 diet. As shown in Supplementary Fig. S5, the affinity of 1,25(OH)_2_D_3_ for Vdr (R270L) is somewhat lower than that of 25(OH)D_3_. Based on the increased plasma level of 25(OH)D_3_ (F(1, 11) = 74.0, *p* < 0.001) and the decreased plasma level of 1,25(OH)_2_D_3_ (F(1, 12) = 22.8, *p* < 0.001) in the *Vdr* (R270L) rats after 25(OH)D_3_ treatment, 25(OH)D_3_ was considered to be a major ligand for Vdr (R270L) in these rats. The significantly higher levels of 24,25(OH)_2_D_3_ (F(1, 11) = 52.2, *p* < 0.001) and 24-oxo-25(OH)D_3_ (F(1, 11) = 38.4, *p* < 0.001) strongly suggest the induction of *Cyp24a1* expression (F(1, 11) = 27.5, *p* < 0.001). These results strongly suggest that 25(OH)D_3_ binds to Vdr (R270L) to induce the expression of the Cyp24a1 gene.

## Discussion

**Figure 5:**
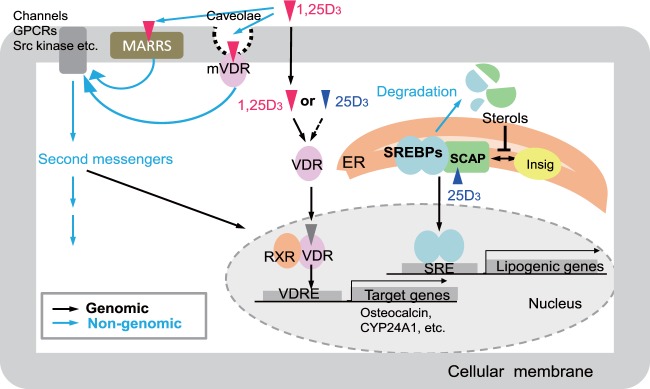
Putative modes of action of vitamin D. Black and blue arrows indicate genomic and nongenomic pathways, respectively. GPCRs, G protein-coupled receptors; MARRS, membrane-associated, rapid response steroid-binding receptor; VDR, vitamin D receptor; mVDR, membrane-bound vitamin D receptor; RXR, retinoid X receptor; VDRE, vitamin D response element; ER, endoplasmic reticulum; SREBPs, sterol regulatory element–binding proteins; SCAP, SREBP cleavage-activating protein; SRE, sterol regulatory element.

The range of vitamin D functions can be elucidated by comparing activities in the GM rats generated in this study (Fig. 5). Previous studies have shown that vitamin D exerts VDR-mediated genomic and nongenomic actions^20,21^ as well as VDR-independent effects^22^. Recently, Asano *et al*.^23^ reported VDR-independent effects of 25(OH)D_3_ on lipid metabolism by inducing degradation of SREBP/SCAP. In addition, ligand-independent effects of VDR have been reported^24,25^. Thus, at least five types of effects of vitamin D and/or the VDR should be considered: (1) VDR-dependent effects of 1,25(OH)_2_D_3_ (VDR-1,25(OH)_2_D_3_)^19,21^, (2) VDR-independent effects of 1,25(OH)_2_D_3_ (non-VDR-1,25(OH)_2_D_3_)^22^, (3) VDR-dependent effects of 25(OH)D_3_ (VDR-25(OH)D_3_)^10^, (4) VDR-independent effects of 25(OH)D_3_ (non-VDR-25(OH)D_3_)^23^, and (5) ligand-independent effects of VDR (VDR-no ligand)^24,25^.

When wild-type and *Vdr* (R270L) rats were compared, a difference was seen in (1) VDR-dependent 1,25(OH)_2_D_3_ effects (Table 2). Similarly, comparisons between *Vdr* (R270L) and *Cyp27b1*-KO rats may reveal (2) VDR-independent effects of 1,25(OH)_2_D_3_. By contrast, comparisons between *Vdr* (R270L) and *Vdr-*KO rats may reveal (3) VDR-dependent actions of 25(OH)D_3_ or (5) ligand-independent effects of the VDR. Thus, *Vdr* (R270L) rats were crucial to this study.

**Table 2:** Putative vitamin D signals including canonical (VDR-1,25(OH)_2_D_3_) and non-canonical actions.

GM strain	Mode of action of vitamin D				
	(1)	(2)	(3)	(4)	(5)
	Vdr-1,25(OH)_2_D_3_	non Vdr -1,25(OH)_2_D_3_	Vdr-25(OH)D_3_	non Vdr -25(OH)D_3_	Vdr -no ligand
WT	Yes	Yes	Yes	Yes	Yes
*Vdr* (R270L)	No*	Yes	Yes	Yes	Yes
*Cyp27b1*-KO	No	No	Yes	Yes	Yes
*Vdr*-KO	No	Yes	No	Yes	No

^*^Based on the affinity of Vdr (R270L) for 25(OH)D_3_ and 1,25(OH)_2_D_3_ and their plasma concentrations in the Vdr (R270L) rats, the Vdr (R270L)-dependent action of 1,25(OH)_2_D_3_ could be negligible compared with the Vdr (R270L)-dependent action of 25(OH)D_3_.

### Comparisons of *Vdr* (R270L) rats with WT rats and the effects of 25(OH)D_3_ administration on *Vdr* (R270L) rats

Analyses using chimeric enzymes in which the ligand-binding domain of Vdr was inserted between split-type luciferases^26^ demonstrated that the affinity of 25(OH)D_3_ for Vdr (R270L) is higher than that of 1,25(OH)_2_D_3_. In the current study, hypocalcemia, elevated parathyroid hormone (PTH), and rickets were observed in mutant *Vdr* (R270L) rats and may have resulted from reduced affinity of 1,25(OH)_2_D_3_ for the variant Vdr. The affinity of 1,25(OH)_2_D_3_ for Vdr (R270L) appeared to be less than 0.1% of that for wild-type Vdr (see Supplementary Fig. S5). Although the plasma 1,25(OH)_2_D_3_ level in *Vdr* (R270L) rats was much higher (approximately 1,100 pg/mL) than that in wild-type rats (24.8 pg/mL), the effects of 1,25(OH)_2_D_3_ mediated by Vdr (R270L) may be quite small. In other words, the effects of a strong hormone with high affinity for the Vdr (i.e., the affinity of 1,25(OH)_2_D_3_ for wild-type Vdr) were lost in *Vdr* (R270L) rats (Table 2). It is worth noting that VDR-mediated effects in the plasma membrane, as proposed by Mizwicki and Norman^21^, may also have been reduced. Therefore, decreased plasma calcium contents, elevation of PTH levels, and osteodysplasia observed in *Vdr* (R270L) rats were likely due to a loss of Vdr-dependent effects of 1,25(OH)_2_D_3_ (Table 2). In contrast, the Vdr-independent effect of 1,25(OH)_2_D_3_ was enhanced by elevated plasma levels of 1,25(OH)_2_D_3_ (50 times higher than wild-type levels). Administration of 25(OH)D_3_ to *Vdr* (R270L) rats normalized osteogenesis and plasma levels of both calcium and PTH. The plasma level of 1,25(OH)_2_D_3_ was dramatically reduced, probably by the reduction in Cyp27b1 expression resulting from the decrease in plasma PTH content.

Overall, our results strongly suggest that the VDR-dependent effects of 1,25(OH)_2_D_3_ (high-affinity ligand of the VDR) are complemented by high levels of low-affinity VDR ligands. The remarkable effects of 25(OH)D_3_ administration on rickets symptoms in *Vdr* (R270L) rats indicate that 25(OH)D_3_ may be efficacious in the treatment of patients with type II rickets caused by the human VDR mutant (R274L).

### Comparison of *Cyp27b1*-KO rats with *Vdr* (R270L) rats and the effects of 25(OH)D_3_ administration on *Cyp27b1*-KO rats

Growth failure and rickets were observed in both *Vdr* (R270L) and *Cyp27b1*-KO rats. In addition, CT analysis revealed morphological changes in cortical bone and trabecular hyperplasia toward the femur. The morphological abnormalities observed in *Cyp27b1*-KO rats appeared to be closely linked with a decrease in cortical bone density and an increase in femoral cancellous bone density. Comparisons of growth rates and plasma calcium levels revealed more severe symptoms in *Cyp27b1*-KO rats than in *Vdr* (R270L) rats. Based on the data in Table 2, the difference between the two strains resulted from the presence or absence of Vdr-independent effects of 1,25(OH)_2_D_3_. It is most likely that *Cyp27b1*-KO rats showed more severe rickets symptoms than mutant *Vdr* (R270L) rats because of the absence of Vdr-independent effects of 1,25(OH)_2_D_3_.

Membrane-associated rapid response steroid-binding proteins, such as GRP58, ERp57, ERp60, and Pdia3^22^, could be involved in calcium absorption in the small intestine, and it is presumed that the remarkable difference in the plasma calcium concentration between *Vdr* (R270L) rats and *Cyp27b1*-KO rats was based on the Vdr-independent effects of 1,25(OH)_2_D_3_. As with *Vdr* (R270L) rats, administration of 25(OH)D_3_ exerted pronounced effects on *Cyp27b1*-KO rats, resulting in normalization of plasma calcium and PTH levels, osteogenesis, and infertility in females. However, 1,25(OH)_2_D_3_ was detected in the blood of *Cyp27b1*-KO rats at the same levels as in wild-type rats. As described in our previous study, the generation of 1,25(OH)_2_D_3_ following 25(OH)D_3_ administration was also observed in *Cyp27b1*-KO mice^12^. 1α-Hydroxylase activity toward 25(OH)D_3_ in a liver mitochondrial fraction prepared from *Cyp27b1*-KO rats suggested that Cyp27a1, a 1α-hydroxylase abundant in the liver, is most likely responsible for the generation of 1,25(OH)_2_D_3_.

It should be noted that 25(OH)D_3_ administration is highly effective in type I rickets models, such as *Cyp27b1*-KO rats. Because human CYP27A1 is capable of converting 25(OH)D_3_ to 1,25(OH)_2_D_3,_ similar effects might be expected in humans.

### Comparison of *Vdr* (R270L) rats and *Vdr*-KO rats

#### Osteogenesis

Growth rates slower than those of wild-type rats were observed in both *Vdr* (R270L) rats and *Vdr*-KO rats. Abnormal osteogenesis and rupture of the growth plate of the epiphyseal cartilage were also seen in both GM rat strains. Toluidine blue staining indicated that disorganized cartilaginous growth plates were observed in both *Vdr* (R270L) rats and *Vdr*-KO rats (Fig. 2a), whereas Goldner staining indicated that increased unmineralized osteoids were seen in *Vdr* (R270L) rats but not in *Vdr*-KO rats (see Supplementary Fig. S9). These results may suggest that *Vdr* (R270L) rats show features of both rickets and osteomalacia but *Vdr*-KO rats show only rickets features at 15 weeks of age, although further analysis is needed to determine if the *Vdr*-KO rats indeed do not show osteomalacic features at this age.

#### Comparison of calcium and PTH contents

The plasma calcium level in *Vdr*-KO rats at 15 weeks did not differ significantly from that in wild-type rats. However, the plasma PTH level in *Vdr*-KO rats was much higher than that in wild-type rats. These results suggest that *Vdr*-KO rats appear to cause tertiary hyperparathyroidism. This type of hyperparathyroidism occurs as a result of long-term secondary hyperparathyroidism. The more complete loss of signaling via Vdr in the *Vdr*-KO rats than in the *Vdr* (R270L) rats likely led to more severe hypocalcemia early on, to higher PTH levels (Fig. 2d) and then to tertiary hyperparathyroidism, which normalized the serum calcium level and resulted in calcifications, such renal stones, as shown in Fig. 2f^27^. In fact, the parathyroid gland in *Vdr*-KO rats was larger than that in wild-type rats (data not shown). In contrast, *Vdr* (R270L) rats showed a slightly lower plasma calcium concentration than wild-type rats.

#### Comparison of skin and hair

Quite abnormal skin and alopecia were seen in *Vdr*-KO rats at 25 weeks (see Supplementary Fig. S7) but not in *Vdr* (R270L) rats. Alopecia was also observed in *Vdr*-KO mice and humans. Several reports have proposed that non-ligand-mediated effects of the VDR are required to maintain the normal hair cycle^16,24,25,28^. However, based on the data in Table 2, the possibility of a role for the absence of Vdr-dependent 25(OH)D_3_ effects in these phenotypes cannot be discarded. To demonstrate differences between ligand-independent effects of the Vdr and Vdr- dependent 25(OH)D_3_ effects, the mutant Vdr, which cannot bind any natural vitamin D derivatives, might be useful.

### Application of GM rats to the development of vitamin D analogs

Several thousand vitamin D analogs have been synthesized, and many have been studied in clinical trials, including those for treating type I rickets, osteoporosis, psoriasis, renal osteodystrophy, leukemia, and pancreatic, prostate, and breast cancers^11,29–31^. However, none have been approved for cancer treatment. 1,25(OH)_2_D_3_ and its analogs actually induce differentiation and control tumor cell proliferation through the VDR-dependent phosphatidylinositol 3-kinase pathway^32^ and by suppressing IL-12 secretion^33^, which is VDR independent. Inhibition of angiogenesis is also an important anticancer mechanism of some vitamin D analogs. However, the precise anticancer mechanism, which may include VDR-dependent and VDR-independent pathways, is not fully understood. Our system using GM rats could be useful to reveal the VDR-dependent and/or VDR-independent mechanisms of such therapeutic approaches, facilitating clinical applications.

## Materials and Methods

### Materials

25(OH)D_3_ was kindly provided by DSM (Limburg, Holland). 1,25(OH)_2_D_3_ was not detected in the 25(OH)D_3_ by LC-MS/MS analysis, indicating that the content of 1,25(OH)_2_D_3_ was less than 0.00003%^12^. [26,27-Methyl-^2^H6]-25(OH)D_3_ ([^2^H6]-25(OH)D_3_) was synthesized in Okano’s laboratory^34^. HPLC-grade organic solvents were purchased from Nacalai Tesque (Kyoto, Japan) and Wako Pure Chemicals (Osaka, Japan). Authentic standards of 24*R*,25(OH)_2_D_3_, and 24-oxo-25(OH)D_3_ were prepared as previously described^35^. Other chemicals were commercially available and of the highest quality.

### Animals and diets

Jcl:Wistar rats were obtained from CLEA Japan Inc. (Tokyo, Japan). Embryonic microinjection for genome editing was performed by KAC Co., Ltd. (Kyoto, Japan).

The generated GM rats were kept at room temperature (22 to 26 °C) and in 50 to 55% humidity with a 12 h light/dark cycle. They were allowed food and water *ad libitum* and fed a CE-2 formula diet (see Supplementary Table S4, CLEA Japan, Inc., Tokyo, Japan) containing 1.15% calcium and 2,100 IU vitamin D3/kg diet. The *Vdr* (R270L) and *Vdr-*KO rats for analysis were fed an F-2 formula diet (see Supplementary Table S5, Oriental Yeast Co., Tokyo, Japan) containing 0.74% calcium and 2000 IU vitamin D/kg diet^12^ after weaning because the CE-2 diet partially reversed their rickets symptoms. By contrast, the *Cyp27b1*-KO rat**s** for analysis were continuously fed the CE-2 diet because most male KO rats were not able to survive more than 10 weeks when fed the F-2 diet (data not shown). Homozygotes of all GM strains (*Cyp27b1*-KO, *Vdr* (R270L),and *Vdr-*KO) were maintained by mating of heterozygotes. Genotypes of each strain were determined by electrophoresis of PCR products of the target site for *Cyp27b1*-KO or direct sequencing for *Vdr* (R270L) and *Vdr*-KO (see Supplementary Figs. S1 and S2).

All experimental protocols using animals were performed in accordance with the Guidelines for Animal Experiments at Toyama Prefectural University and were approved by the Animal Research and Ethics Committee of Toyama Prefectural University.

### Generation of *Cyp27b1*-KO, *Vd*r (R270L), and *Vdr*-KO rats by the CRISPR/Cas9 genome editing system

Three strains of genetically modified rats were generated as described in the Supplementary Methods. Briefly, they were generated by the CRISPR/Cas9 genome editing system. The target site for *Cyp27b1*-KO was selected to delete the cysteine at position 462 in exon 8, which is the 5th ligand of heme iron and an active center of Cyp27b1 (see Supplementary Fig. S1a). The target site for mutant *Vdr* (R270L) was selected to disrupt the array near the arginine codon (CGC) at position 270 of the *Vdr* gene (see Supplementary Fig. S2a).

### Validation of off-target sites

Potential off target sites (OTSs) of *Cyp27b1* and *Vdr* in rat genomes were searched by the CRISPR Direct tool (http://crispr.dbcls.jp/) to validate the off-target events. Potential off-target sites were analyzed when the sequence was the same as 12 subsequent nucleotides from the protospacer adjacent motif (PAM) of the target sequence (see Supplementary Figs. S3 and S4). The OTS regions were amplified, purified, and then directly sequenced.

### 25(OH)D_3_ treatment for *Cyp27b1*-KO and *Vdr* (R270L) rats

*Cyp27b1-*KO rats and *Vdr* (R270L) rats were fed 25(OH)D_3_ for comparison of its effects on rickets symptoms. A 25D-F-2 pellet diet containing 1.5 mg 25(OH)D_3_ per 1 kg F-2 was prepared by Oriental Yeast Co. During 25D-F-2 feeding, the average daily food intake was 18.6 ± 3.7 g in *Cyp27b1*-KO rats and 18.1 ± 0.7 g in *Vdr* (R270L) rats, whereas the body weight of *Cyp27b1*-KO rats was significantly lower than that of *Vdr* (R270L) rats. Consequently, the food intake per kg body weight was calculated to be 113.2 ± 16.9 g/kg bw/day in *Cyp27b1*-KO rats and 56.0 ± 6.4 g/kg bw/day in *Vdr* (R270L) rats. Thus, the dose of 25(OH)D_3_ was calculated to be 168.9 ± 25.1 μg/kg bw/day in *Cyp27b1*-KO rats and 80.9 ± 7.7 μg/kg bw/day in *Vdr* (R270L) rats (the values are shown as the means ± SEMs (n = 2–3 animals/group)). GM rats were fed 25D-F-2 after 5 weeks of age and mated with their respective genotypes. The effects of 25(OH)D_3_ administration beyond generations were examined by continuous feeding of 25D-F-2 as described previously^12^. Briefly, the dams were fed 25D-F-2 continuously after birth of offspring until weaning, and the offspring were also fed 25D-F-2 until 15 weeks of age.

### Measurement of plasma 25(OH)D_3,_ 24,25(OH)_2_D_3_ and 24-oxo-25(OH)D_3_ concentrations by LC/MS/MS analysis

Plasma concentrations of 25(OH)D_3,_ 24,25(OH)_2_D_3_, and 24-oxo-25(OH)D_3_ were measured by using a modified LC-APCI-MS/MS method^12^. The method involves the use of deuterated 25(OH)D_3_ (*d*_6_-25(OH)D_3_) as an internal standard compound and the selection of a precursor and product ion with an MS/MS multiple reaction monitoring (MRM) method. Briefly, the internal standard *d*_6_-25(OH)D_3_ (0.5 ng/10 μL) was added to plasma (40 μL) and precipitated with acetonitrile (200 μL). The supernatant was evaporated, and the residue was dissolved with ethyl acetate (400 μL) and distilled water (200 μL). After vigorous shaking, the ethyl acetate phase was removed and evaporated. Extracted vitamin D metabolites from plasma were derivatized by 4-[2-(6,7-dimethoxy-4-methyl-3-oxo-3,4-dihydroquinoxalyl)ethyl]-1,2,4-triazoline-3,5-dione (DMEQ-TAD) to obtain high sensitivity by increasing ionization efficiency^36^. Separation was carried out using a reverse-phase C_18_ analytical column (CAPCELL PAK C_18_ UG120, 5 μm; (4.6 I.D. × 250 mm) (SHISEIDO, Tokyo, Japan) with a solvent system consisting of (A) acetonitrile, (B) distilled water (0–5 min A = 30%, 5–34 min (A) = 30 → 70%, and 34–37 min (A) = 70 → 100%) as the mobile phase and a flow rate of 1.0 mL/min. All MS data were collected in the positive ion mode, and quantitative analysis was carried out using MS/MS-MRM of the precursor/product ion for DMEQ-TAD-25(OH)D_3_ (*m*/*z*: 746.5/468.1), DMEQ-TAD-24,25(OH)_2_D_3_ (m/z: 762.5/468.1), DMEQ-TAD-24-oxo-25(OH)D_3_ (*m*/*z*: 760.5/468.1), and DMEQ-TAD-*d*_6_-25(OH)D_3_ (*m*/*z*: 752.5/468.1) with a dwell time of 200 ms. The values of the coefficient of variation (CV) for the intra-assay and inter-assay variation were 6.5 and 1.4% in the measurement of 25(OH)D_3_, 11.6 to 9.5% in the measurement of 24,25(OH)_2_D_3,_ and 6.4 and 5.3% in the measurement of 24-oxo-25(OH)D_3_, respectively.

### Measurement of plasma 1,25(OH)_2_D_3_ with an ELISA kit

The plasma concentration of 1,25(OH)_2_D_3_ was measured using a 1,25-(OH)_2_ Vitamin D ELISA Kit (Immundiagnostik, Bensheim, Germany) as described previously^12^. Prior to the assay, solid phase extraction using Choromabond XTR (Immundiagnostik, Bensheim, Germany) and a Sep-pak Silica Cartridge (Waters, MA, U.S.A.) was performed according to the manufacturer’s protocol.

### Measurement of bone mineral density

Bone mineral density (BMD) was determined between the proximal and distal epiphysis of the left femur. After muscle removal, the left femora of the rats (n = 4–5 animals for each group) were scanned using an X-ray CT system (Latheta LCT-200; Hitachi Aloka Medical, Tokyo, Japan). The parameters used for the CT scans were as follows: tube voltage, 50 kV; tube current, 500 μA; integration time, 3.6 ms; axial field of view, 48 mm; and isotropic voxel size, 48 μm. The mineral content of the femur was calculated using LaTheta software (Hitachi Aloka Medical). A threshold density of 160 mg/cm^3^ was selected to distinguish mineralized from unmineralized tissue. The density range was calibrated daily with a manufacturer-supplied standard^20^.

### Histological analysis

Von Kossa staining was performed to detect the calcification of the femur. Toluidine blue staining was also performed to analyze the structure of the epiphyseal growth plate in the femur, which is formed with the cartilage layer and is involved in the longitudinal growth of long bones. Villanueva Goldner staining was conducted to detect unmineralized osteoids in cortical and cancellous bone of the femur. These procedures were performed by Kureha Special Laboratory Co., Ltd. (Fukushima, Japan). Hematoxylin and eosin (H&E) staining of dorsal skin was performed to examine the details of skin disorders in *Vdr*-KO rats.

### Real-time quantitative PCR

Total RNA of the rat kidney and intestines was isolated using Isogen II (Nippon Gene, Tokyo, Japan). cDNA synthesis and real-time PCR were performed as described previously^11^. The renal mRNA expression of *Cyp24a1* (GenBank accession number, NM_201635; forward primer, 5′-AGCCCGGGGCAGATTTCCTCTG-3′; reverse primer, 5′-CATATTCCTCAGGTCTTCCGC-3′) was determined by the ΔΔCt method using rat *β-actin* (GenBank accession number, NM_031144; forward primer, 5′-AGGCCCAGAGCAAGAGAGGCAT-3′, reverse primer, 5′-CATATCGTCCCAGTTGGTGACA-3′) as a control.

### Measurement of plasma calcium, phosphorus, and parathyroid hormone (PTH) concentrations

The plasma calcium and phosphorus concentrations were measured using a Calcium E-Test Wako Kit (Wako Pure Chemical, Osaka, Japan) and Phospha C-Test Wako Kit (Wako Pure Chemical), respectively. The plasma PTH concentration was determined using a Rat Intact PTH ELISA Kit (Immutopics Inc., San Clemente, CA, U.S.A.).

### Preparation of liver mitochondrial and microsomal fractions and measurement of the 1α-hydroxylation activity of 25(OH)D_3_ in each fraction

Liver mitochondrial and microsomal fractions were prepared from *Cyp27b1*-KO rats using the same methods as described in our previous study^12^. The mitochondrial fraction was incubated in 100 mM Tris-HCl buffer (pH 7.4) containing 5000 nM ADX, 500 nM ADR, 10 μM 25(OH)D_3_, and 1 mM NADPH at 37 °C for 1 h. The microsomal fraction was incubated in 100 mM phosphate buffer (pH 7.4) containing 10 μM 25(OH)D_3_ and 1 mM NADPH at 37 °C for 1 h. 25(OH)D_3_ and its metabolites in each fraction were applied to reverse-phase HPLC, and the fractions around the retention time of 1,25(OH)_2_D_3_ were collected using the same methods as described in our previous study^12^. The isolated fractions were further subjected to normal-phase HPLC under the same conditions as described in our previous study^12^, and the fractions around the retention time of 1,25(OH)_2_D_3_ were collected and dried. The resultant residue was derivatized and analyzed by LC-APCI-MS/MS according to the method in section 8, except for the detection of the precursor/product ion for DMEQ-TAD-1,25(OH)_2_D_3_ (m/z: 762.4/484.0).

### Statistical analysis

Analysis was conducted with the use of IBM SPSS Statistics software (version 25). Student’s t-test was performed to assess the differences in bone mineral density and plasma Ca, PTH and 1,25(OH)_2_D_3_ levels in GM rats. Two-way ANOVA was performed for the analysis of the bone mineral density and levels of plasma Ca, PTH, 1,25(OH)_2_D_3_, 25(OH)D_3_ and its metabolites in *Cyp27b1-*KO and mutant *Vdr* (R270L) rats fed the F-2 or 25D-F-2 diet. Differences were considered significant at *p* < 0.05.

## Supplementary information

Suppl. Information.
