# Supplementary material for: Generation of novel genetically modified rats to reveal the molecular mechanisms of vitamin D actions
Source: Sci Rep. 2020 Mar 30;10:5677. doi: 10.1038/s41598-020-62048-1 (PMC7105495; doi:10.1038/s41598-020-62048-1)
Supplement: Supplementary file 1 — Suppl. Information. [file 41598_2020_62048_MOESM1_ESM.docx]

**Generation of novel genetically modified rats to reveal the molecular mechanisms of vitamin D actions**

Miyu Nishikawa^1^, Kaori Yasuda^2^, Masashi Takamatsu^1^, Keisuke Abe^2^, Kairi Okamoto^2^, Kyohei Horibe^2^, Hiroki Mano^2^, Kimie Nakagawa^3^, Naoko Tsugawa^4^, Yoshihisa Hirota^5^, Tetsuhiro Horie^6^, Eiichi Hinoi^6,7^, Toshio Okano^3^, Shinichi Ikushiro^1^, Toshiyuki Sakaki^2^*.

1.Department of Biotechnology, Faculty of Engineering, Toyama Prefectural University, 5180 Kurokawa, Imizu, Toyama 939-0398, Japan.

2.Department of Pharmaceutical Engineering, Faculty of Engineering, Toyama

Prefectural University, 5180 Kurokawa, Imizu, Toyama 939-0398, Japan.

3.Department of Hygienic Sciences, Kobe Pharmaceutical University, 4-19-1 Motoyamakita-machi, Higashinada-ku, Kobe 658-8558, Japan.

4.Department of Health and Nutrition, Faculty of Health and Nutrition, Osaka Shoin Women’s University, 4-2-26 Hishiya-nishi, Higashi-Osaka 577-8550, Japan

5.Laboratory of Biochemistry, Faculty of Bioscience and Engineering, College of Systems Engineering and Science, Shibaura Institute of Technology, 307 Fukasaku, Minuma-ku, Saitama 337-8570, Japan.

6.Laboratory of Pharmacology, Department of Bioactive Molecules, Gifu Pharmaceutical University, Gifu, Japan

7.United Graduate School of Drug Discovery and Medical Information Sciences, Gifu University, Gifu, Japan*.*

Running head: Generation of novel GM rats to reveal vitamin D actions

Address correspondence to : 5180 Kurokawa, Imizu, Toyama 939-0398, Japan. Fax: +81-766-56-2498; E-mail: [tsakaki@pu-toyama.ac.jp](mailto:tsakaki@pu-toyama.ac.jp)

Key words: genome editing, CRISPR/Cas9, vitamin D, 25-hydroxyvitamin D_3_,

cytochrome P450, CYP27B1, vitamin D receptor, rickets,

,

**Supplemental methods.**

**1. Preparation of Cas9 mRNA, single guide (sg) RNAs, and single-stranded oligo DNA nucleotides (ssODNs) for genome editing by the CRISPR/Cas9 system**

Cas9 mRNA was obtained from Apro Science (Naruto, Japan). To design sgRNAs, genomic sequences of rat *Cyp27b1* (NC_005106.4) and *Vdr* (NC_005101.4) were submitted to the CRISPR Direct tool (<http://crispr.dbcls.jp/>). SgRNAs and ssODNs were obtained from Fasmac (Atsugi, Japan).

**2. Microinjections into rat embryos**

Female rats were superovulated by a single intraperitoneal injection of pregnant mare serum gonadotropin (Aska Pharmaceutical, Tokyo, Japan), followed by a single intraperitoneal injection of human chorionic gonadotropin (Aska Pharmaceutical). Superovulated females were cohabited with male rats, and pronuclear-stage embryos were collected from the females on the following day.

To edit the *Cyp27b1* gene, a mixture of 25 ng/µL sgRNA 1, 25 ng/µL sgRNA 2, and 50 ng/µL Cas9 mRNA was microinjected into the pronuclei of embryos. To edit the *Vdr* gene, a mixture of 25 ng/µL sgRNA, 100 ng/µL Cas9 mRNA, and 50 ng/µL ssODN was microinjected into the pronuclei of embryos. The microinjected embryos were then transferred into pseudopregnant rats.

**3.** **Founder generation**

Pups delivered from the transferred embryos were obtained by caesarean section on the day of birth and nursed by foster mothers. After weaning, tail tips were biopsied for mutation analysis. Crude genomic DNA was extracted from the tail tips by Lyppo (Wako Pure Chemicals, Osaka, Japan). Polymerase chain reaction (PCR) was performed using KOD Fx Neo (Toyobo, Osaka, Japan) with specific primer sets (Supplementary Table 3). PCR products were purified using a FastGene™ Gel/PCR Extraction Kit (Nippon Gene, Tokyo, Japan) according to the manufacturer’s protocol and directly sequenced and analyzed on an Applied Biosystems 3.500 DNA sequencer (Thermo Fisher Scientific, Waltham, MA, USA) using a BigDye Terminator v3.1 Cycle Sequencing Kit (Thermo Fisher Scientific).

**4. Measurement of the affinity of VDR ligands for wild-type rat Vdr or its mutant, Vdr (R270L)**

　The pET-11d-N-6xHN-LucC-LBD(WT)-LucN and pET-11d-N-6xHN-LucC-LBD(R270L)-LucN vectors were transformed into *E. coli* strain BL21(DE3) (BioDynamics Laboratory Inc., Japan) according to the manufacturer’s instructions. A single colony from a fresh plate was picked and grown at 37 °C in 2 mL of LB medium containing 100 mg/mL ampicillin [LB(Amp)]. An overnight culture of these cells was used to inoculate 500 mL of LB(Amp). Protein expression was induced at an OD_600_ of 0.4 with 0.1 mM isopropyl-1-thio-β-D-galactopyranoside [IPTG (Nacalai Tesque, Inc., Japan)], followed by incubation for an additional 3 h at 15 °C. Cells were pelleted by centrifugation at 5,000 x *g* at 4 °C for 10 min, resuspended in suspension buffer [25 mM Tris-HCl (pH 7.4), 10 mM DTT and protease inhibitor cocktail (Nacalai Tesque, Inc., Japan)] and lysed by sonication for 15 s × 7. The lysate was centrifuged at 20,000 × *g* for 30 min at 4 °C. The lysates containing the LucC-LBD(WT)-LucN and LucC-LBD(R270L)-LucN proteins were used for the *in vitro* luciferase complementation assay.

　The *E. coli* lysate containing the LucC-LBD(WT)-LucN biosensor or LucC-LBD(R270L)-LucN biosensor proteins was diluted with 2 mg/mL BSA solution, and the total volume was adjusted to 50 μl using reaction solution [25 mM Tris-HCl (pH 7.4), 10 mM DTT, and 0.1 mg/mL BSA]. A total of 50 μl of the reaction solution containing 0.033 μl of LucC-LBD(WT)-LucN biosensor lysate or 0.02 μl of LucC-LBD(R270L)-LucN biosensor lysate was plated into a 96-well plate within 2-3 min. Next, EtOH-dissolved **25(OH)D_3_ or 1α,25(OH)_2_D_3_** was added to the well at 0-10.000 nM and incubated at room temperature (23-26 °C) (preincubation) for 30 min. As **25(OH)D_3_** and **1α,25(OH)_2_D_3_** were dissolved in EtOH at a final concentration of 1%, wells containing 1% EtOH were used as controls. Then, 50 μl of luciferin solution [25 mM Tris-HCl (pH 7.4), 20 mM MgSO_4_, 2 mM D-Luciferin (Thermo Scientific, CA, U.S.A.) and 4 mM ATP] was injected into the 96-well plate. After the addition of luciferin solution**, the light intensity of each well was measured by a** 96-microplate **luminometer (**photon detection**) at 30 min.** In this study, the relative light intensity in the presence of **25(OH)D_3_** or **1α,25(OH)_2_D_3_** was calculated in comparison with the light intensity in the absence of **25(OH)D_3_** or **1,25(OH)_2_D_3_** (1% EtOH) as a negative control.

**5. Western blot analysis.**

The kidney was homogenized with RIPA buffer containing 1% protease inhibitor cocktail (Nacalai Tesque, Kyoto, Japan). After incubation for 30 min on ice, the homogenate was centrifuged at 10,000 ×g for 20 min. The supernatant was additionally centrifuged at 105,000 ×g for 60 min. The resultant supernatant was collected as the cytosolic fraction and used for Western blot analysis. The total protein concentration was determined by BCA protein assay. Twenty or sixty micrograms of protein was separated by SDS-PAGE and electrically transferred onto a PVDF membrane (GE Healthcare, Buckinghamshire, UK). To detect the rVDR proteins, the membranes were incubated with a mouse anti-VDR (1:2000, Santa Cruz, CA, U.S.A.) antibody in Can Get Signal solution (Toyobo, Osaka, Japan) at 4°C for 12 h and then reacted with HRP-conjugated secondary horse anti-mouse IgG (H+L) (1:5,000, Cell Signaling, CA, U.S.A.). The immobilized proteins were visualized using an ECL prime Western blotting reagent (GE Healthcare, Buckinghamshire, UK). The chemiluminescence signals were detected using a ChemiDoc imaging system (Bio-Rad, CA, U.S.A. ).

**6.** **Measurement of plasma creatinine**

Plasma concentrations of creatinine were determined using a LabAssay™ Creatinine Kit (Wako Pure Chemicals) according to the manufacturer’s protocol.

**7.** **V. Goldner staining**

V. Goldner staining of femur specimens was performed by Kureha Special Laboratory (Iwaki, Japan).


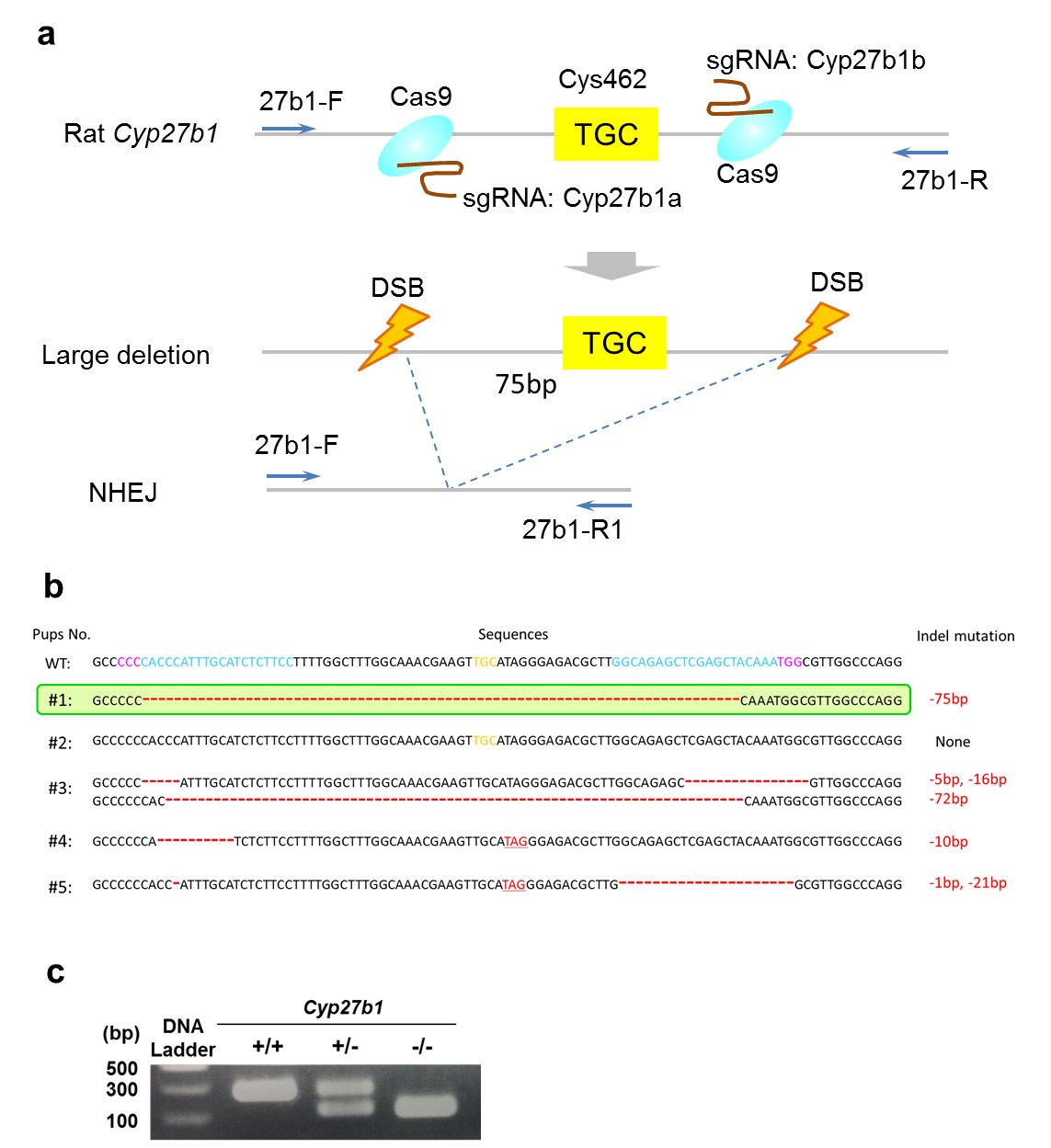


**Supplementary Fig. S1. Generation of *Cyp27b1*-KO rats by CRISPR/Cas9 genome editing.**

1. Scheme of *Cyp27b1* deletion in founder #1. 27b1-F and 27b1-R, primers for genotyping; DSB, double-strand break. (b) In-del mutations in *Cyp27b1*-KO founders induced by genome editing. Magenta, protospacer adjacent motif (PAM) sequence; cyan, target site for sgRNA; yellow; codon for Cys462; red, in-del mutation; red with an underline, stop codon produced by a frameshift triggered by in-del mutation. (c) Electrophoresis profiles of polymerase chain reaction amplicons for the *Cyp27b1* target region.


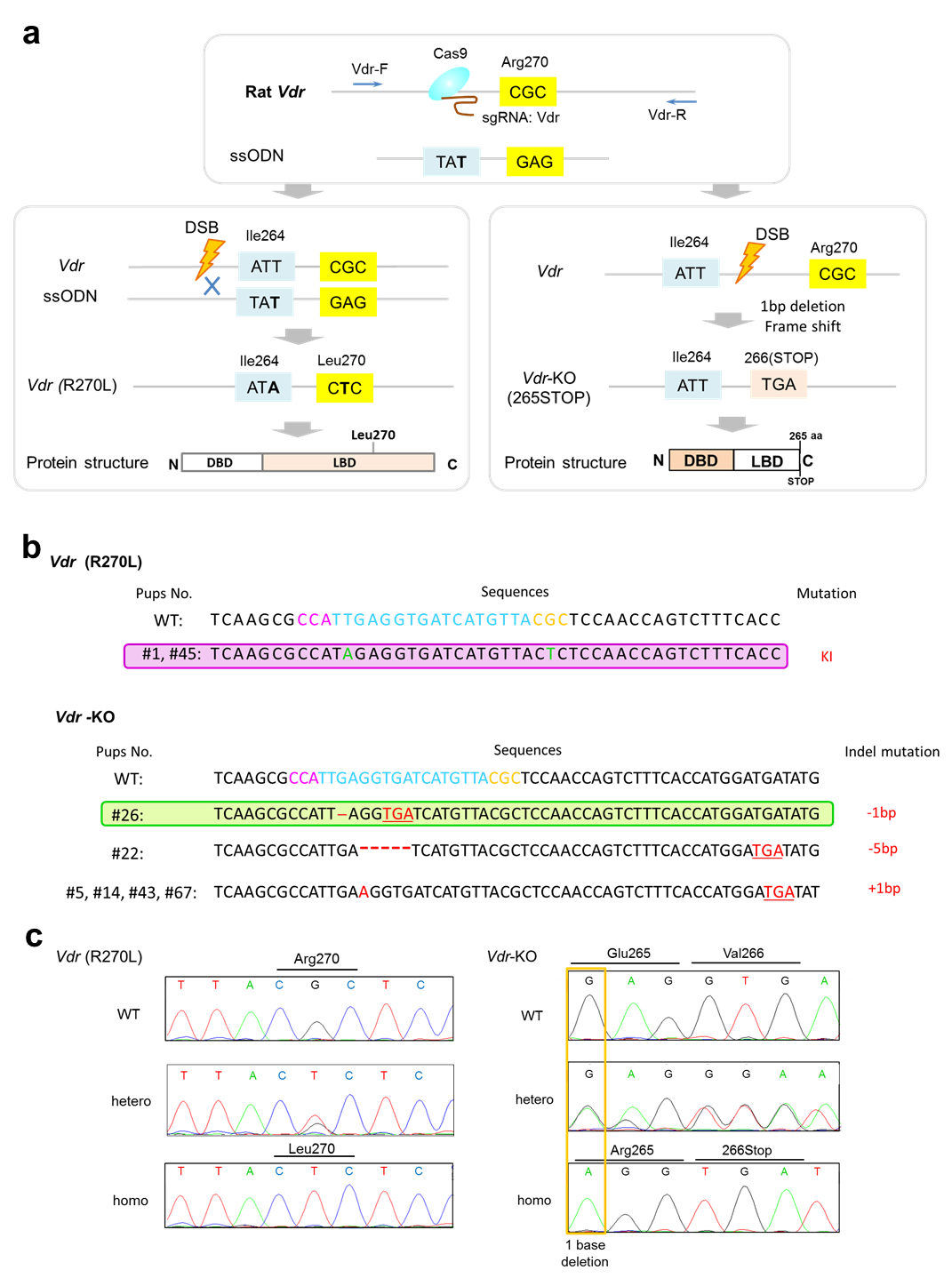


**Supplementary Fig. S2. Generation of Vdr (R270L) and *Vdr*-KO rats by CRISPR/Cas9 genome editing.**

(a) Scheme of *Vdr* mutation or deletion. Vdr-F and Vdr-R, primers for genotyping; DSB, double-strand break. DBD, DNA-binding domain; LBD, ligand-binding domain. (b) In-del mutations in *Vdr* (R270L) and *Vdr*-KO founders induced by genome editing. Magenta, protospacer adjacent motif (PAM) sequence; cyan, target site for sgRNA; yellow; codon for Cys462; red, in-del mutation; red with an underline, stop codon produced by a frameshift triggered by in-del mutation. (c) Sequence profiles of the *Vdr* target region in *Vdr* (R270L) and *Vdr*-KO rats.

**
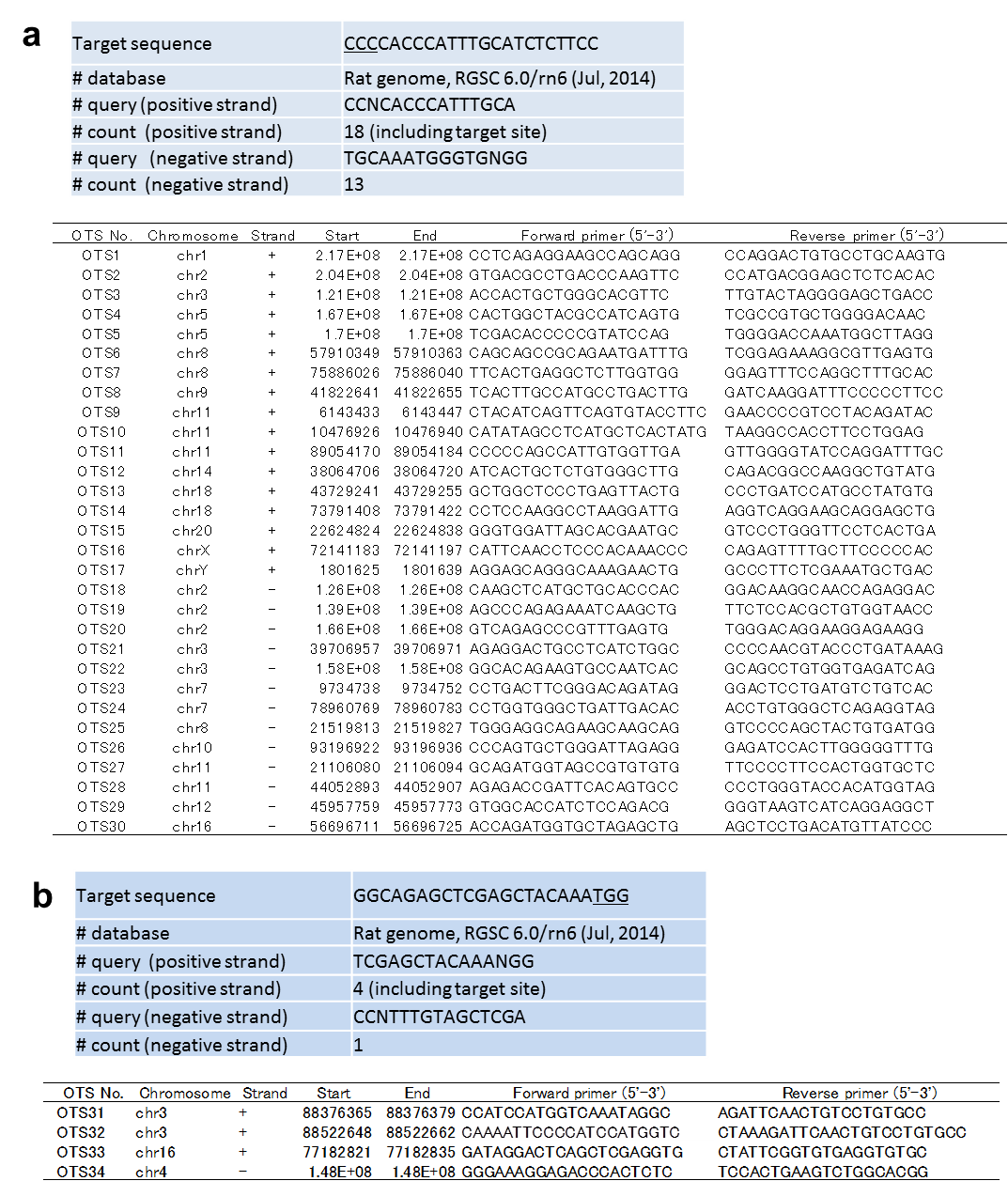
**

**Supplementary Fig. S3. Potential off-target cleavage sites** (**OTSs) of *Cyp27b1* in the rat genome**

Potential OTSs of *Cyp27b1* target site 1 (a) and target site 2 (b) and primer sequences for OTS analysis. The forward or reverse primer was used for direct sequencing.


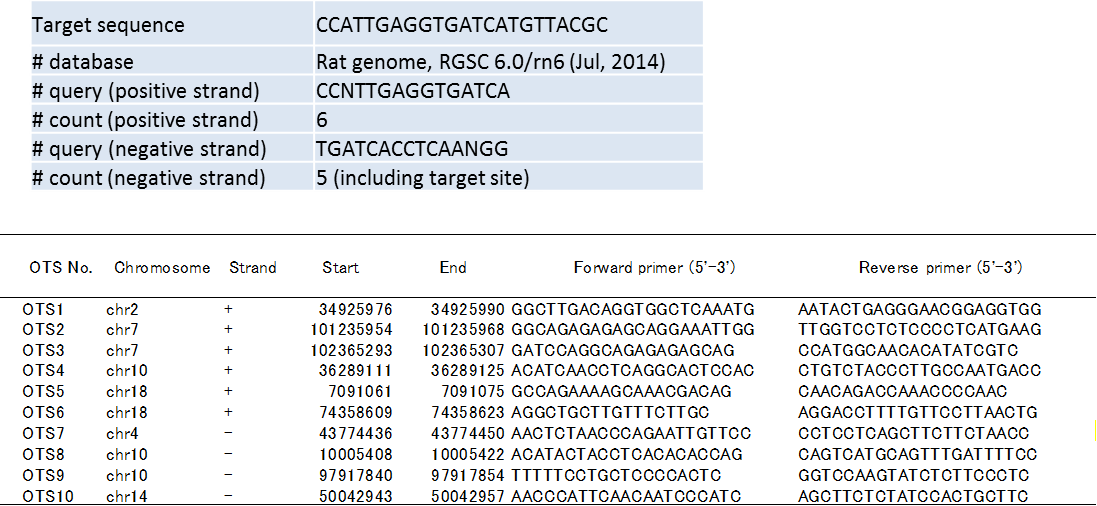


**Supplementary Fig. S4. Potential OTSs of *Vdr* in the rat genome**

Potential OTSs of the *Vdr* target site and primer sequences for OTS analysis. The forward or reverse primer was used for direct sequencing.

**
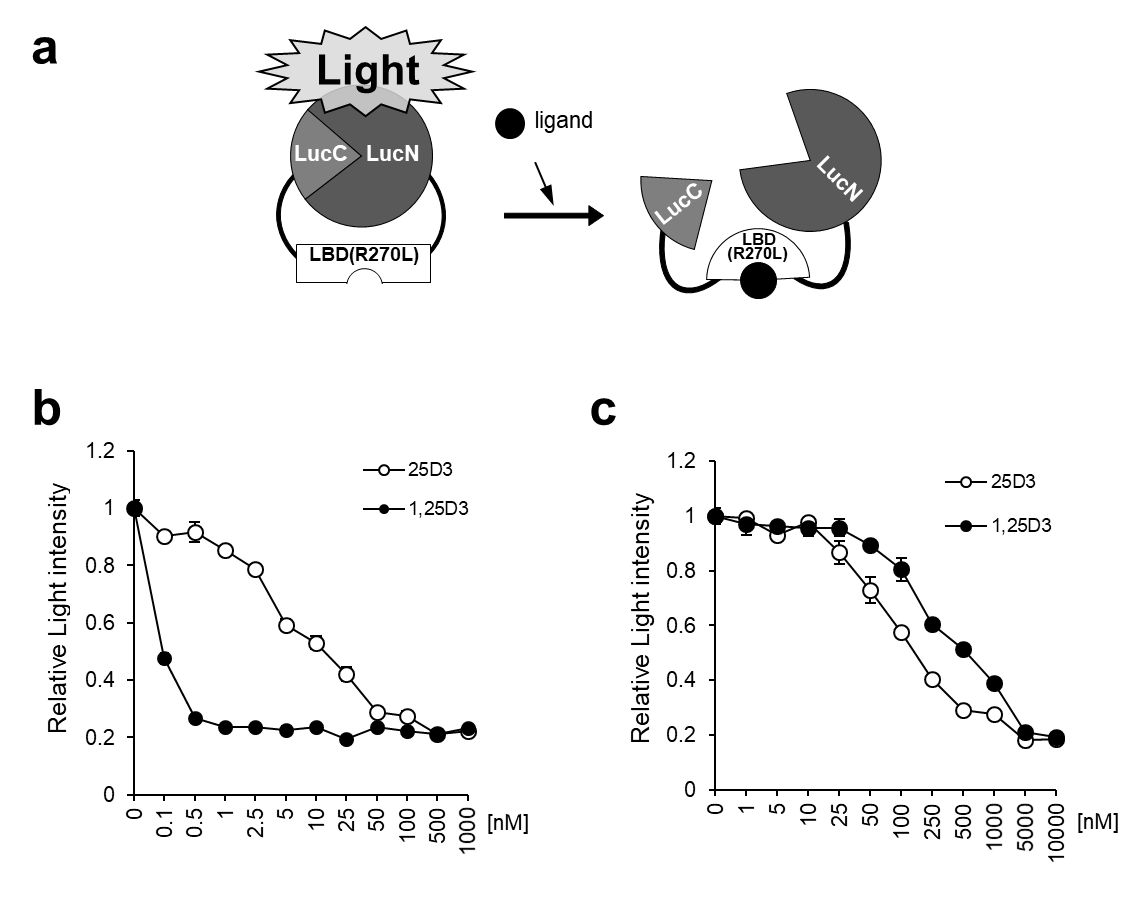
 Supplementary Fig. S5. Scheme of the split luciferase-based ligand-binding assay (a) and ligand-binding assay using a chimeric fusion protein of firefly luciferase (Luc) with the ligand-binding domain (LBD) of wild-type Vdr (b) or Vdr (R270L) (c).**

Binding of the VDR ligand to the LucC-LBD(R270L)-LucN biosensor may cause a conformational change in LBD (R270L) that leads to disruption of the functional complex between the LucN and LucC fragments of the split luciferase.

The LucC-LBD(WT)-LucN or LucC-LBD(R270L)-LucN biosensor proteins were treated with various concentrations (0 to 10,000 nM) of 1,25(OH)_2_D_3_ or **25(OH)D_3_ f**or 30 min. The relative light intensity compared to that of the control (0 nM = 1% EtOH) is shown. Data are represented as the means ± SEMs, *n* = 3.

1,25(OH)_2_D_3_ showed a much higher affinity for wild-type Vdr than 25(OH)D_3_.

In contrast, 1,25(OH)_2_D_3_ showed a somewhat lower affinity for Vdr (R270L) than 25(OH)D_3_.

**
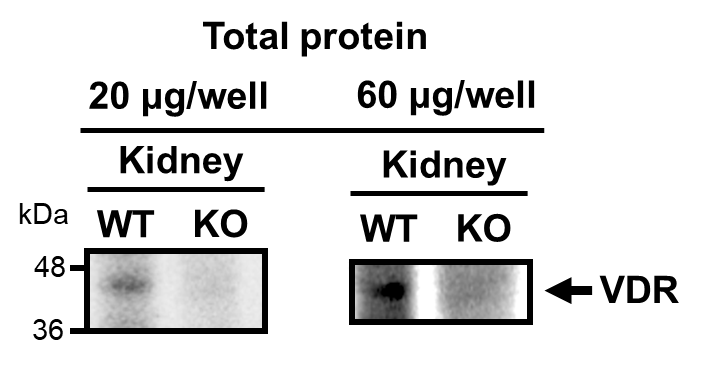
**

**Supplementary Fig. S6.** **Western blotting analysis of Vdr proteins in the kidney prepared from WT or *Vdr*-KO rats.** Total proteins (20 or 60 μg) containing Vdr were separated by SDS-PAGE. The location of protein size markers (kDa) is shown on the left. The black arrow indicates the specific band of Vdr proteins.


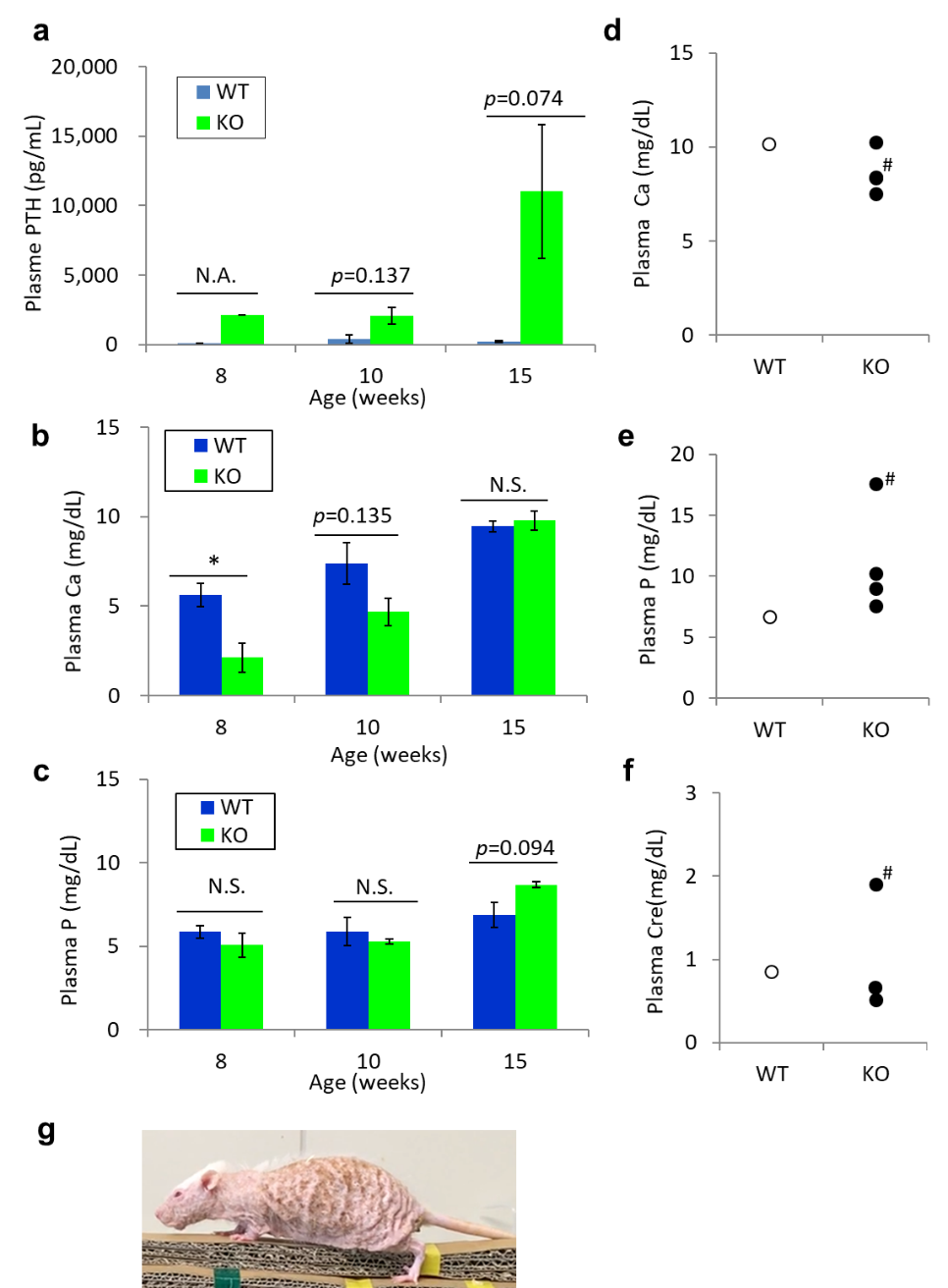


**Supplementary Fig. S7. Age-dependent disorder of calcium and phosphorus homeostasis and skin phenotypes in *Vdr-*KO rats.**

Plasma concentrations of (a) parathyroid hormone (PTH), (b) calcium, and (c) phosphorus in *Vdr*-KO rats at 8, 10, and 15 weeks of age. Values are shown as the means ± SEMs (n = 1-6 animals/group for PTH, n = 2-6 animals/group for calcium and phosphorus). N.A.: Not analyzed statistically because the wild-type (WT) values were from one animal. N.S: not significant. **p* < 0.05 by Student’s t-test. (d-f) Plasma concentrations of (d) calcium, (e) phosphorus, and (f) creatinine in *Vdr*-KO rats at 25 weeks of age. Skin phenotypes of *Vdr*-KO rats at 25 weeks of age (g). Values derived from individual animals are shown as open (WT) or closed (KO) circles. The values were not analyzed statistically. The source of values with “#” was the animal in Fig. 3f.


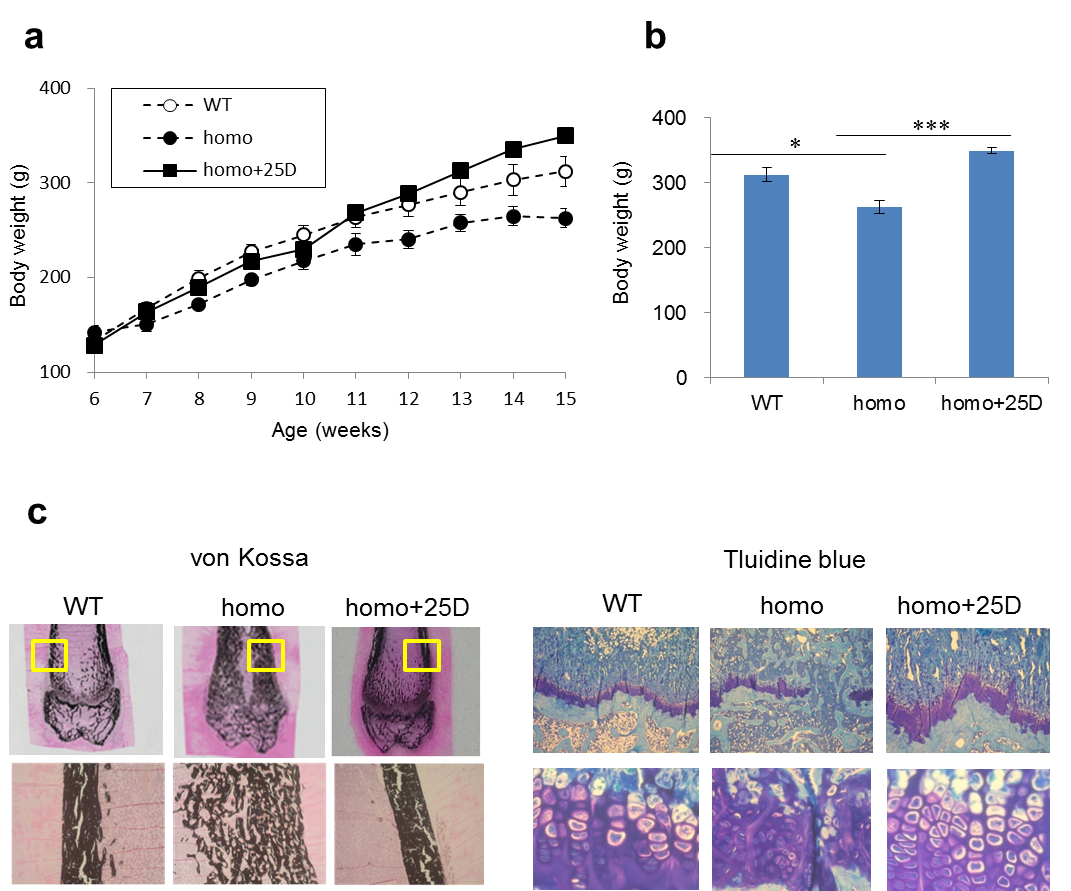


**Supplementary Fig. S8. Effects of 25(OH)D_3_ on growth and osteogenesis in *Cyp27b1-*KO rats.**

(a) Growth curve of *Cyp27b1*-KO rats treated with or without 25(OH)D_3_. Values are shown as the means ± SEMs (n = 2-6). (b) Body weights at 15 weeks of age. Values are shown as the means ± SEMs (n = 2-6). (c) Histological images of the femur. von Kossa staining of the distal femur (left panels) and toluidine blue staining of the epiphyseal plate (right panels) were performed. *p < 0.05 and ***p < 0.001 by a two-way analysis of variance.

**Supplementary Fig. S9.**　**Villanueva Goldner staining of femora of WT rats, *Cyp27b1*-KO rats, *Cyp27b1-KO* rats fed 25D3 diet, *Vdr (*R270L) rats, and *Vdr*-KO rats.**

Upper panels, diaphysis; middle panels, distal epiphysis; and lower panels, proximal epiphysis.

**Supplementary Fig. S10.** **Conversion of 25(OH)D_3_ to 1,25(OH)_2_D_3_ in the liver mitochondrial fraction of *Cyp27b1*-KO homozygote rats with (lower) or without (upper) fadrozole.**

Each arrow indicates the 6*R*- and 6*S*- isomers of DMEQ-TAD adducts of 1,25(OH)_2_D_3_ [12].

**Supplementary Table S1. OTS validation of *Cyp27b1* in rat genome.**

NA: not analyzed.

4 base del: 4 base deletion. This mismatch was also observed in *Vdr*-R270L, *Vdr*-KO, and WT rats.

**Supplementary Table S2. OTS validation of *Vdr* in rat genome.**


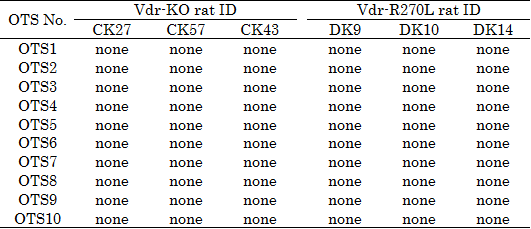


**Supplementary Table S3. Primer sequences for genotyping of GM rats.**

**Supplementary Table S4. Composition of CE-2 formula diet.**

**Supplementary Table S5. Composition of F-2 formula diet.**
